# Physiological uptake in FDG PET simulating disease

**DOI:** 10.2349/biij.2.4.e59

**Published:** 2006-10-01

**Authors:** S Ahmad Sarji

**Affiliations:** Department of Biomedical Imaging, Faculty of Medicine, University of Malaya, Kuala Lumpur, Malaysia

**Keywords:** FDG PET, physiological uptake, pitfalls, artefacts

## Abstract

Many potential pitfalls and artefacts have been described in PET imaging that uses F-18 fluorodeoxyglucose (FDG). Normal uptake of FDG occurs in many sites of the body and may cause confusion in interpretation particularly in oncology imaging. Clinical correlation, awareness of the areas of normal uptake of FDG in the body and knowledge of variation in uptake as well as benign processes that are FDG avid are necessary to avoid potential pitfalls in image interpretation. In this context, optimum preparation of patients for their scans can be instituted in an attempt to reduce the problem. Many of the problems and pitfalls associated with areas of normal uptake of FDG can be solved by using PET CT imaging. PET CT imaging has the ability to correctly attribute FDG activity to a structurally normal organ on CT. However, the development of combined PET CT scanners also comes with its own specific problems related to the combined PET CT technique. These include misregistration artefacts due to respiration and the presence of high density substances which may lead to artefactual overestimation of activity if CT data are used for attenuation correction.

## INTRODUCTION

F-18 fluorodeoxyglucose (FDG) is the radiotracer most commonly used for PET imaging. The molecule is easiest to understand by interpreting it backwards, starting with the molecule of glucose. The deoxy part implies cleavage of a hydroxyl group from the glucose. The attachment of the F-18 tracer to the glucose replaces the hydroxyl group.

**Figure 1:**
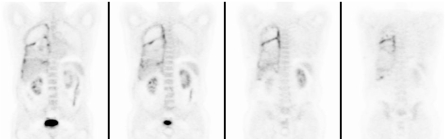
FDG PET scan (coronal view) in a patient who had undergone pleurodesis. Increased activity is seen along the pleura and fissure in the right hemithorax [Image courtesy of Centre for Molecular Imaging, Peter MacCallum Cancer Centre, East Melbourne, Victoria, Australia].

In this way, the FDG molecule acts like glucose during initial enzymatic reactions within cells, but the altered structure prevents further metabolism. This essentially traps FDG within cells and FDG accumulates in most tissues at a rate proportional to glycolysis [1]. Malignant cells have increased glucose transporter proteins on their cell surface as well as enhanced rates of glycolysis. The enhanced glycolytic rate of malignant cells facilitates their detection utilizing PET FDG imaging. It is this attribute that causes metabolically active tumours to appear ‘hot’ on PET scans. PET measures FDG retention per volume of tissue. Some tumours are known to have a high retention of FDG while others have variable retention of FDG. Unfortunately, FDG is not a cancer-specific agent and its uptake has been described in a number of inflammatory lesions including sarcoidosis, tuberculosis, fungal infection, and cerebral abscess. The increased accumulation is probably related to a markedly increased rate of glycolysis within activated inflammatory cells [2,3,4]. Talc pleurodesis produces a visceral and parietal pleural granulomatous inflammation. Increased FDG activity has been reported in sites of pleural talc up to three years following the procedure, presumably secondary to pleural inflammation (Figure 1) [5, 6].

Many potential pitfalls and artefacts associated with FDG PET imaging have been described. It is important that we learn about these pitfalls and recognise the important areas of normal uptake of FDG or absence of uptake that may be of no significance. This is necessary so that patients can be optimally prepared for their scans and accurate interpretation can be made.

## UPTAKE OF FDG IN THE HEAD AND NECK REGION

**Figure 2:**
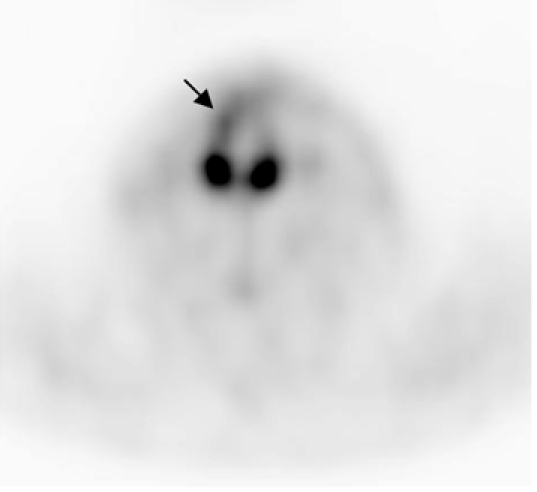
FDG PET scan (axial view) through the larynx. There is asymmetric uptake of tracer seen in the laryngeal muscles while slightly more intense uptake is seen on the right side in this patient with left laryngeal nerve palsy (arrow) [Image courtesy of Centre for Molecular Imaging, Peter MacCallum Cancer Centre, East Melbourne, Victoria, Australia].

In the brain, very intense tracer uptake occurs in the normal cerebral cortex and basal ganglia, glucose being the predominant substrate for brain metabolism. The total uptake in the brain is approximately 6 % of the injected dose. Normal lymphatic tissue may display low to moderate FDG uptake in the head and neck region. This is seen in the lingual and palatine tonsils and at the base of the tongue because of physiologic activity associated with the lymphatic tissue in Waldeyer’s ring. Symmetry is helpful in evaluating FDG uptake in the head and neck. Uptake should be symmetrical in the palatine and lingual tonsils. The soft palate can also show tracer uptake. Variable, but typically low, uptake can be seen in the salivary glands. The larynx can accumulate tracer while the patient is talking. Asymmetric uptake of FDG can be seen in the laryngeal muscles in patients with laryngeal nerve palsy contralateral to the side of the nerve dysfunction and should not be misinterpreted as pathologic (Figure 2).

**Figure 3:**
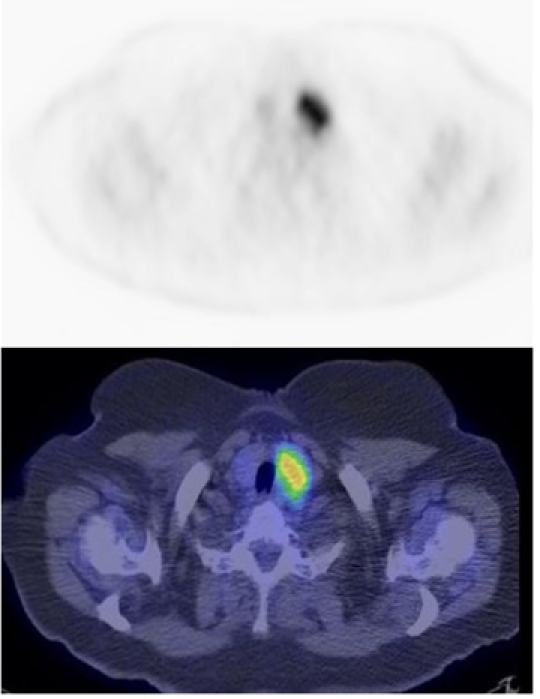
FDG PET and fused PET CT images (axial view) at the level of the thyroid gland. Focal intense uptake of tracer was incidentally found in the left lobe of the thyroid [Image courtesy of Centre for Molecular Imaging, Peter MacCallum Cancer Centre, East Melbourne, Victoria, Australia].

A moderate amount of uptake can be seen in the anterior part of the floor of the mouth due to the genioglossus muscle which prevents the tongue from falling back in patients who are in the supine position. The cervical spinal cord may also demonstrate normal uptake, best seen in sagittal images. Diffuse symmetric uptake can be seen in the normal thyroid gland in about 2% of scans. Diffuse thyroid uptake can occur in association with thyroiditis or Graves' disease. Focal thyroid uptake can occur with autonomously functioning thyroid nodules and thyroid malignancies. Patients with focal uptake should be further evaluated due to a higher risk of the result being associated with malignancy [7, 8] (Figure 3).

## UPTAKE OF FDG IN THE THORAX, ABDOMEN AND PELVIS

Uptake in the thymus is commonly seen in children. However, not all children will have visible thymic activity on FDG PET imaging. The cause of this variability in pediatric thymic accumulation of FDG is unclear, but is likely to be related to physical and emotional stressors which influence thymic metabolism. In general, physiologic uptake of FDG in the thymus disappears in adolescence in conjunction with involution of the thymus.

**Figure 4:**
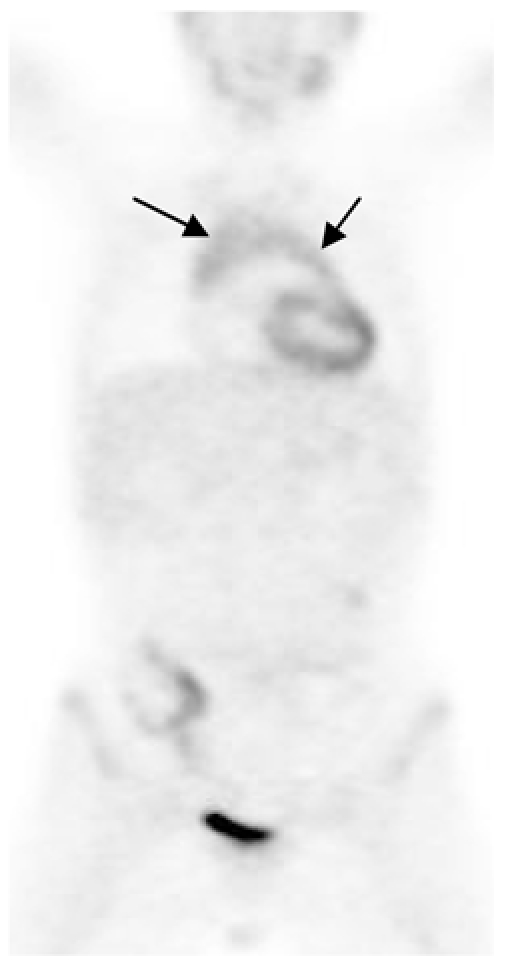
FDG PET scan (coronal view) in a young adult. Normal thymus is seen as an inverted V area of moderate uptake (arrows) [Image courtesy of Centre for Molecular Imaging, Peter MacCallum Cancer Centre, East Melbourne, Victoria, Australia].

Rebound thymic hyperplasia is seen in young patients treated for malignancy. Following chemotherapy, FDG uptake can be seen in the thymus of 75% of children and in 5% to 16% of adults and enlargement can persist for up to six months following completion of chemotherapy. There are several signs to ascertain non-pathologic thymic FDG accumulation. Normal thymic activity will appear triangular or “V” shaped (bilobed) and will usually have low to moderate uptake (Figure 4).

**Figure 5:**
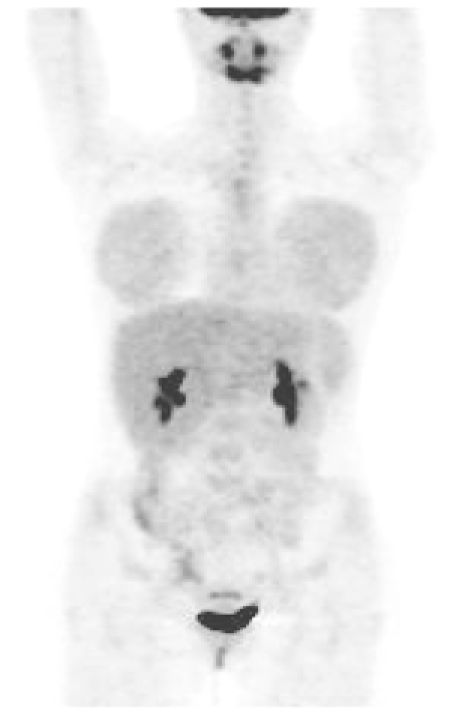
FDG PET scan (coronal view) in a young female. There is bilateral symmetrical moderate uptake of tracer in the breasts [Image courtesy of Centre for Molecular Imaging, Peter MacCallum Cancer Centre, East Melbourne, Victoria, Australia].

Lack of uptake in the pre-therapy scan should be an indicator for post treatment thymic hyperplasia. FDG accumulation in the thymus suggests pathology when it does not have a typical triangular shape or if the activity is very intense [9,10]. Glandular breast tissue may show moderate uptake of FDG and is relatively increased in pre-menopausal subjects and post-menopausal subjects taking hormone replacement therapy (Figure 5). Marked uptake may be seen in lactating breasts.

Cardiac activity is variable ranging from no discernible activity above the background pool activity to intense activity throughout the left ventricle myocardium. It is unusual to see atrial or right-ventricular activity unless there is cardiac disease affecting those chambers. In the fasting state where insulin levels are low, FDG uptake in cardiac muscle should be low. Myocardial uptake is enhanced in the presence of high blood glucose levels, therefore cardiac activity is marked in the post-prandial state. Little myocardial activity is generally noted in the fasting state as the myocardium preferentially utilises fatty acids for energy generation. However, uptake can be variable even in the fasting state. We may see activity in the aorta and great vessels, particularly in artherosclerotic disease.

**Figure 6:**
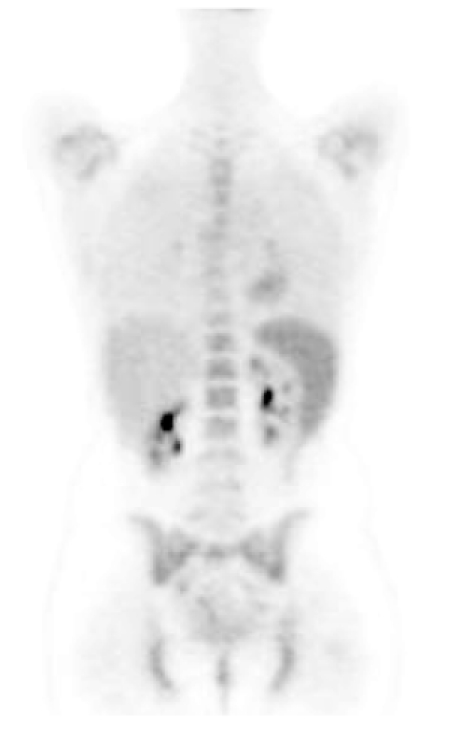
FDG PET scan (coronal view) in a patient who had received growth colony stimulating factor (GCSF). There is increased marrow activity seen in the spine and pelvis and increased splenic activity [Image courtesy of Centre for Molecular Imaging, Peter MacCallum Cancer Centre, East Melbourne, Victoria, Australia].

In the urinary tract, FDG is filtered by the glomerulus and is not reabsorbed by the renal tubules. So, significant activity may be displayed in any part of the urinary tract or surgical urinary diversions such as ileal conduits. The liver, spleen and bone marrow normally show homogenous low grade uptake. Bone marrow and spleen normally show less intense uptake than the liver. However, in patients receiving growth colony stimulating factor (GCSF), increased marrow and splenic activity are shown on FDG PET scans (Figure 6).

Uptake in the gastrointestinal tract can be highly variable. The esophagus does not usually show significant activity. However, the gastroesophageal junction may show normal uptake. Homogenous low uptake within the stomach wall is relatively common. If the stomach is contracted, this may appear as a round focal area of moderate activity. Small intestinal uptake is variable and usually of low grade. Colonic activity may be quite marked, particularly in the caecum and rectosigmoid junction. In general, uptake is highest in the colon, followed by the small bowel, with the stomach showing uptake of lowest intensity. Bowel uptake can be diffuse but not focal.

**Figure 7:**
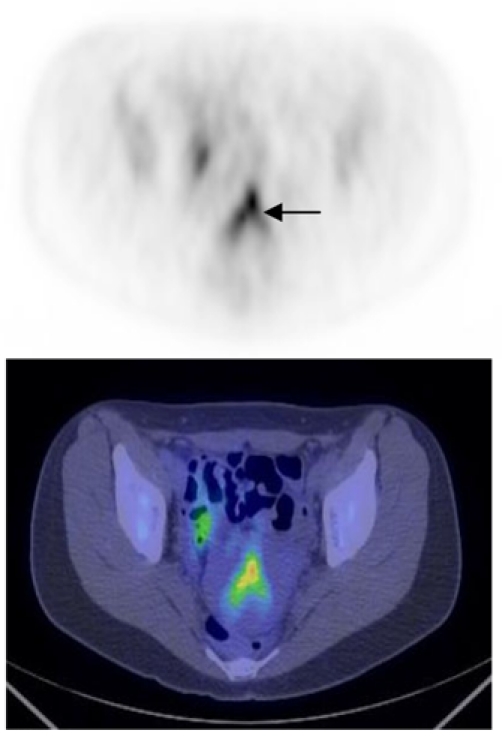
FDG PET CT scan (axial view) of the pelvis. Activity is seen in the endometrial cavity in this patient who was menstruating (arrow). On the fused image, the uterus is seen to be retroverted [Image courtesy of Centre for Molecular Imaging, Peter MacCallum Cancer Centre, East Melbourne, Victoria, Australia].

Testicular uptake in a male is normally seen and is symmetrically diffuse. However, in the female, ovarian uptake is not usually seen. If ovarian uptake is seen in a post-menopausal patient, malignancy must be ruled out. Faint uterine activity is common. Uptake in the uterus has been reported during menstruation and ovulation in pre-menopausal women and in relation to fibroids, but in practice it is an uncommon finding (Figure 7).

## UPTAKE OF FDG IN SOFT TISSUES

**Figure 8:**
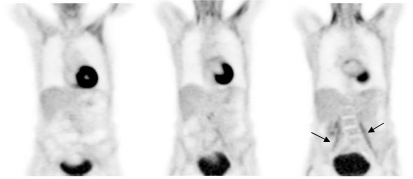
FDG PET scan (coronal view).There is increased activity seen in the neck and shoulder muscles. Moderate uptake is also seen in the psoas muscles in this patient with scoliosis (arrows) [Image courtesy of Centre for Molecular Imaging, Peter MacCallum Cancer Centre, East Melbourne, Victoria, Australia].

A common area for interpretative pitfall is related to FDG uptake in active skeletal muscle. In relaxed and rested patients, no significant skeletal muscle uptake is noted. Muscular imbalance, e.g., post surgery, scoliosis, may result in increase FDG uptake in affected muscles (Figure 8) .Most skeletal muscle activity can easily be recognised as such.

**Figure 9:**
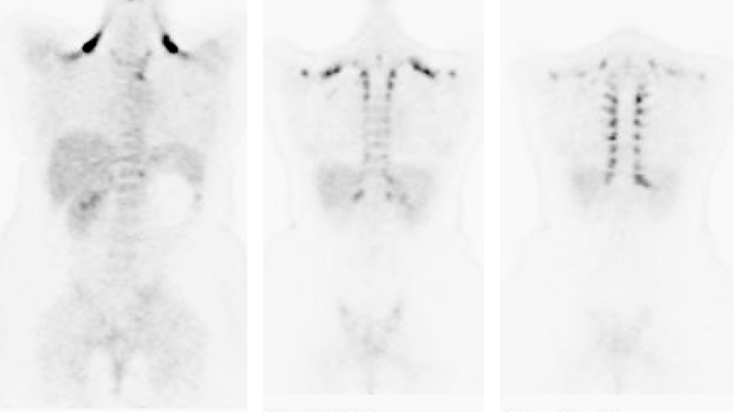
FDG PET scan (coronal view). Intense tracer uptake is seen into brown fat seen in the supraclavicular, intercostal and paravertebral regions [Image courtesy of Centre for Molecular Imaging, Peter MacCallum Cancer Centre, East Melbourne, Victoria, Australia].

**Figure 10:**
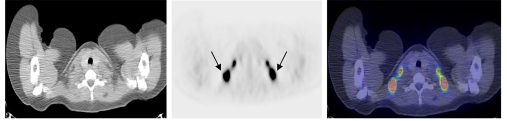
From left to right - axial CT image, PET image and fused PET CT image at the same level showing areas of intense uptake fat in the supraclavicular regions (arrows). The PET/CT fused images facilitated differentiation between fat and pathological tissue [Image courtesy of Centre for Molecular Imaging, Peter MacCallum Cancer Centre, East Melbourne, Victoria, Australia].

Prominent tracer uptake has also been described within the supraclavicular fat on FDG PET scans in about 2% to 4% of patients. The aetiology is not well understood, but is felt to be related to the presence of “brown fat” (brown adipose tissue). Brown fat is most prominent in newborns and diminishes with age. Unlike white adipose tissue it has the capacity to generate heat. It is stimulated by several factors, including exposure to cold. The incidence of tracer uptake in brown fat also increases in women. Areas in which prominent tracer uptake into brown fat is seen are in the supraclavicular regions followed by the axillae, mediastinum, intercostal, paravertebral, and perinephric regions (Figure 9). Even when recognised as a benign variant, the degree of uptake can easily obscure malignant lymphadenopathy in the region. With PET CT imaging it is now easier to differentiate fat and pathological tissue (Figure 10) [11].

It is essential to be aware of the clinical correlation of other sites of uptake or absence of uptake that may be of no significance. These include healing fractures (less than three months) and healing surgical incision sites, sites of previous radiation therapy (no or low uptake), joint prosthesis (not infected), degenerative joint disease, stoma sites (colostomy, ileostomy, tracheostomy), chest tube drainage site, biopsy sites and porta-cath sites.

## PATIENT PREPARATION AND OTHER TECHNICAL ISSUES

Adequate patient preparation is necessary to minimise the appearance of potential artefactual uptake patterns that make interpretation difficult. Exercise should be avoided on the day of scanning to avoid muscle uptake. Patients need to be totally relaxed and kept warm. If the patient is cold and nervous, clenching their hands will increase muscle activity in the forearms, resulting in increased tracer uptake. Stress-induced muscle tension is often seen in the trapezius and paraspinal muscles. Those known to have muscle spasms may be administered benzodiazepines before the FDG injection. This minimises uptake by normal skeletal muscles, particularly the proximal muscles, which would make neck and supraclavicular nodal evaluation difficult. Patients are advised to avoid talking, chewing and swallowing too much to reduce accumulation of tracer into muscles of mastication and the larynx. Brown fat has the capacity to generate heat. It is known to increase glucose uptake when the sympathetic nervous system is activated by cold stimulation. Therefore, keeping the patient warm may be helpful in reducing uptake into brown fat.

Patients are prepared for PET CT scans in the same manner as for PET scanning. The CT scan is done before the PET scan. For oncological indications, an extended body survey is usually acquired, typically including images from the skull base to the proximal thighs. The typical acquisition time is less than a minute for the CT scan. The CT component is performed as a non contrast low radiation dose scan. It is performed primarily for attenuation correction and anatomical correlation. Next, without the patient moving or changing position, a PET scan encompassing the same imaging field is performed. The resulting PET CT studies are interpreted at a computer workstation with dedicated software allowing review of attenuation corrected PET, CT and fusion PET CT images in three orthogonal planes. Both the CT and PET CT fusion images are used to localise PET uptake abnormalities. Many of the problems with regard to pitfalls and areas of normal uptake described above are solved with the use of PET CT imaging. PET CT imaging will reduce diagnostic uncertainty with respect to physiologic activity by allowing more confident interpretation related to areas of anatomically normal structures. It merges anatomic and molecular data with the aim of producing one integrated diagnosis.

Development of the combined PET CT scanners comes with its own specific problems related to the combined PET CT technique [8]. Differences in breathing patterns between the CT and the PET scans may lead to misregistration, e.g., a pulmonary nodule situated in the periphery and in the bases of the lungs. Misregistration may be minimised by performing the CT during expiration instead of inspiration. High density substances, e.g., contrast agents or metallic objects, can lead to artefactual overestimation of activity if CT data are used for attenuation correction. The artefacts can be recognised by studying the uncorrected image data. Use of intravenous contrast during the CT acquisition may lead to over-correction of attenuation artefactual hot areas in the attenuation corrected image and quantitative over-estimation of FDG activity/uptake. A separate contrast-enhanced CT examination is indicated if use of intravenous contrast is essential to increase diagnostic accuracy.

In summary;

- FDG will localise to any part of the body where there is high physiologic activity.
- Basic to the proper interpretation of PET CT scans is a clear understanding of the normal variants of uptake and awareness of the benign processes that are FDG avid to avoid potential pitfalls in image interpretation.
